# Modularity-Guided Functional Brain Network Analysis for Early-Stage Dementia Identification

**DOI:** 10.3389/fnins.2021.720909

**Published:** 2021-08-05

**Authors:** Yangyang Zhang, Xiao Jiang, Lishan Qiao, Mingxia Liu

**Affiliations:** ^1^School of Mathematics Science, Liaocheng University, Liaocheng, China; ^2^School of Science and Technology, University of Camerino, Camerino, Italy; ^3^Department of Radiology and BRIC, University of North Carolina at Chapel Hill, Chapel Hill, NC, United States

**Keywords:** functional brain network, modularity, feature selection, signed spectral clustering, classification

## Abstract

Function brain network (FBN) analysis has shown great potential in identifying brain diseases, such as Alzheimer's disease (AD) and its prodromal stage, namely mild cognitive impairment (MCI). It is essential to identify discriminative and interpretable features from function brain networks, so as to improve classification performance and help us understand the pathological mechanism of AD-related brain disorders. Previous studies usually extract node statistics or edge weights from FBNs to represent each subject. However, these methods generally ignore the *topological structure* (such as modularity) of FBNs. To address this issue, we propose a modular-LASSO feature selection (MLFS) framework that can explicitly model the modularity information to identify discriminative and interpretable features from FBNs for automated AD/MCI classification. Specifically, the proposed MLFS method first searches the modular structure of FBNs through a signed spectral clustering algorithm, and then selects discriminative features via a modularity-induced group LASSO method, followed by a support vector machine (SVM) for classification. To evaluate the effectiveness of the proposed method, extensive experiments are performed on 563 resting-state functional MRI scans from the public ADNI database to identify subjects with AD/MCI from normal controls and predict the future progress of MCI subjects. Experimental results demonstrate that our method is superior to previous methods in both tasks of AD/MCI identification and MCI conversion prediction, and also helps discover discriminative brain regions and functional connectivities associated with AD.

## 1. Introduction

Resting-state functional magnetic resonance imaging (rs-fMRI) provides a non-invasive measure of brain activity and attracts considerable attention for understanding the brain organization (Bijsterbosch et al., 2017; Zhang et al., 2020). Function brain network (FBN) derived from rs-fMRI scans has been increasingly employed to computer-aided diagnosis of brain disorders, such as autism spectrum disorder (Jie et al., 2018a; Wang et al., 2019a,c; Wen et al., 2019; He et al., 2020), Alzheimer's disease (AD) and its prodromal stage (i.e., mild cognitive impairment, MCI) (Stam, 2014; Fornito et al., 2015; Liu M. et al., 2015; Jie et al., 2018b).

**Figure 1:**
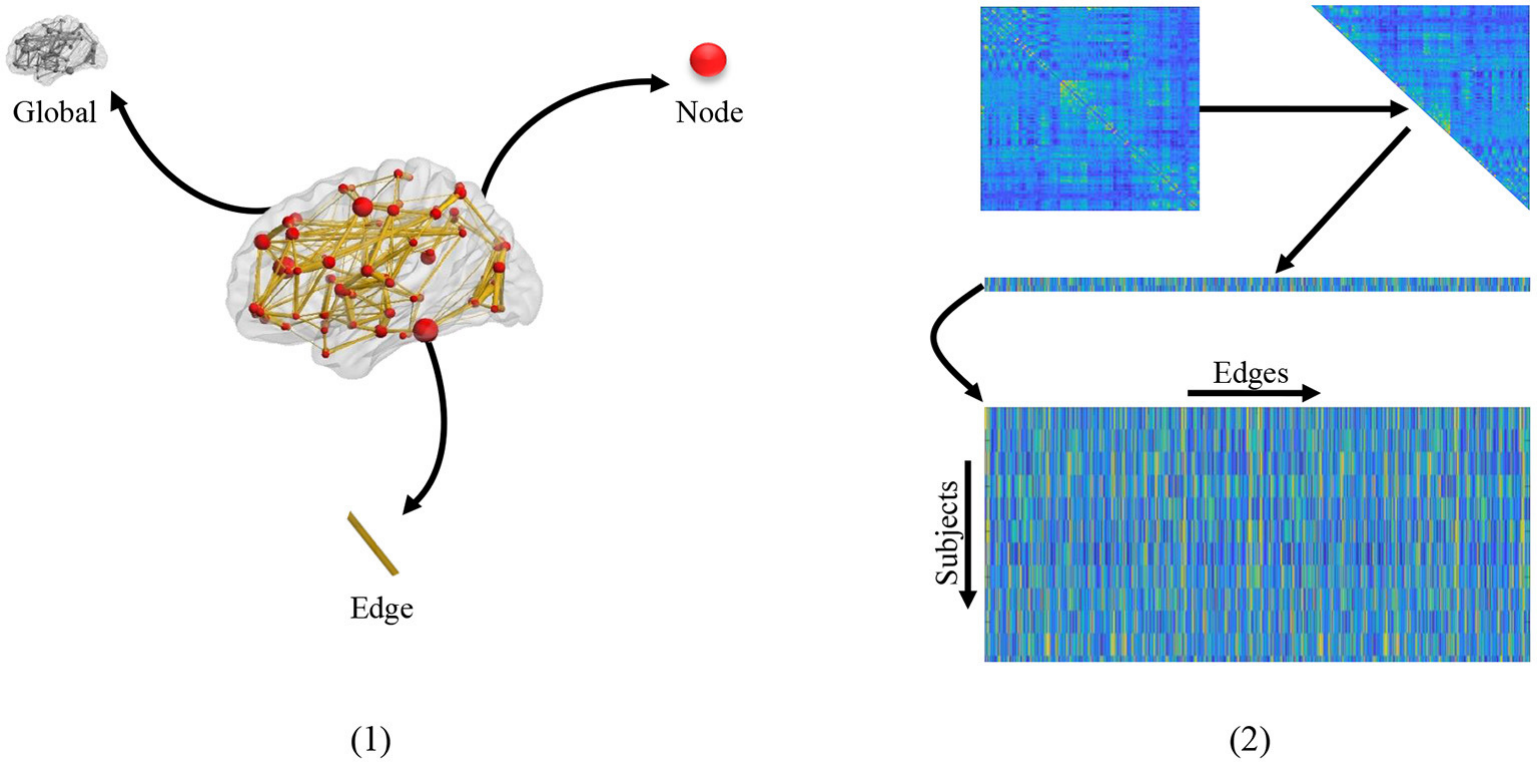
(1) Different granularity of feature. From the clockwise direction is the global-level topology feature, node-level topology feature, and edge-level topology feature, respectively. (2) The mechanism of traditional edge feature extraction in FBN. The network adjacency matrix from each subject is first mapped onto a vector by removing the redundant part if the matrix is symmetric, and then the vectors from all subjects are rearranged together as an input of the following feature selection methods.

Extracting effective features from FBNs is a critical step to improve classification performance and interpretability of brain functional networks (Kim et al., 2019; Qiu et al., 2019). As shown in Figure 1 (1), three kinds of feature representations have been employed for FBN-based disease identification based on different granularities, including global-level topology features, node-level features, and edge-level features. The first one is the global topological statistics of the whole FBN, such as sparsity and efficiency (Hamilton, 2020). Despite its simplicity, the global statistics may lack specificity. That is, due to their global characteristics, the global measures cannot help identify the disease-affected brain regions (i.e., nodes) and functional connections (i.e., edges) in a brain network. The second category focuses on node-based graph statistics (e.g., local clustering coefficients; Wee et al., 2012). They can specifically locate disease-related regions on the node level, but usually fail to recognize the contributions of different edges/connections in a network. Besides, both global- and node-level statistics extracted from FBNs tend to capture different network properties, which requires prior knowledge and thus makes the feature design an intractable problem (Hamilton, 2020).

The third strategy uses edge-level features (e.g., edge weights) to represent a network (Qiao et al., 2017; Xue et al., 2020), which is simple and can naturally obtain the localization of effects on the granularity of edges. In practice, the adjacent matrix of FBN from each subject is generally concatenated into an edge vector (removing the redundant part if the adjacent matrix is symmetric), and then the edge vectors from all subjects are piled up, as shown in Figure 1 (2). In this case, the edge features associated with all subjects are stacked into a matrix for further selection (e.g., through *t*-test and LASSO). However, these methods ignore network topologies such as modularity that provides valuable information for understanding the pathological mechanism of AD-related brain disorders.

Modularity plays an important role in FBN modeling and analysis, and can help us understand operating mechanisms of brain (Shen et al., 2010; Gallen et al., 2016; Wen et al., 2019). Meunier et al. (2009b) conducted FBN analysis and found that FBN has a hierarchical modular organization with a fair degree of similarity between subjects. Motivated by the fact that the brain exhibits a modular organization, we propose a modular-LASSO feature selection (MLFS) framework that consists of a two-step learning scheme. Specifically, the proposed MLFS first searches modular structure of FBNs through a signed spectral clustering algorithm, and then selects discriminative features using group LASSO based on modularity information, followed by a support vector machine (SVM) for brain disease classification. Our proposed method is validated on the public ADNI dataset (Jack et al., 2008) with 563 rs-fMRI scans to identify AD/MCI subjects from normal controls and perform MCI conversion prediction, with experimental results demonstrating its superiority over conventional methods.

The rest of the paper is organized as follows. In section 2, we review the most relevant studies on fMRI-based FBN analysis. In section 3, we introduce the data used in the study and present our method. In section 4, we conduct experiments and provide a comparative evaluation of the involved methods. In section 5, we discuss the impact of parameters, the number of modules, the different node-level features on classification performance and the effect of connectivity variations in FBN, visualize the disease-related features (functional connections) and modules identified by our proposed method, and present limitations of this work as well as future research directions. Finally, we conclude the paper in section 6.

## 2. Related Work

In this section, we briefly review the most relevant studies on feature representation of functional brain networks (FBNs) and existing methods on modularity analysis of FBNs.

### 2.1. Feature Representation of FBNs

As the basis of subsequent classification/regression tasks, feature representation of brain networks is essential for FBN analysis. Currently, three categories of features based on different granularities (global/network-level, node-level, and edge-level) have been employed for representing FBNs.

The first two categories (i.e., global-level and node-level representation) use topological measures to represent the whole brain or brain regions for identifying patients from healthy controls. For example, Feng et al. (2020) extracted spatial and temporal eigenvalue features from high-order dynamic FBNs as feature representations of each subject for AD classification. Jie et al. (2016) extracted local clustering coefficients from hyper-connectivity networks as features to identify subjects with MCI. Although these studies have achieved good results, the topological measures involved in these methods need to be designed manually, which is cumbersome, time-consuming, and also subjective. In the third category, numerous studies represent FBNs by edge-level features (e.g., edge weights) for each subject, followed by edge vector-based feature selection for classification. For example, Sun et al. (2021) extracted edge weight features from sparse FBNs to identify patients with MCI and Autism spectrum disorder (ASD) disorder. Liu F. et al. (2015) extracted connectivity strengths from FBNs as features for social anxiety disorder classification. However, these studies usually ignore the overall topology of functional brain networks (e.g., modularity), and the edge features are generally of large scale, possibly resulting in a series of problems such as the curse of dimensionality and the error of multiple comparisons (Garcia et al., 2017).

### 2.2. Modularity Analysis of FBNs

Previous studies have shown that FBNs exhibit a modular organization, such that they are comprised of a group of sub-networks (Gallen et al., 2016). Research on network modularity helps us to understand the organizational principles of the brain, which has important theoretical significance and practical value in FBN analysis.

Many studies have focused on finding modules in brain networks. For example, Meunier et al. (2009a) studied the modular partitions of resting-state networks in the human brain, and investigated the influence of normal aging on the modular structure. Valencia et al. (2009) investigated modular organization in resting-state networks at the voxel level, and showed modules at a finer grain level. Although these studies on the partition of modules distinguished the different roles and status of nodes, they did not apply the modular structure to the analysis of FBNs (e.g., FBN construction, feature learning, and classification). Recently, many studies applied modularity prior to FBN construction. For example, Qiao et al. (2016) estimated FBNs by incorporating modularity prior, and achieved higher classification accuracy based on the modularized FBNs. Zhou et al. (2018) learned an optimal neighborhood high-order network with sparsity and modularity priors for MCI conversion prediction. However, these existing studies cannot explicitly employ the modular structure to guide the feature selection of brain networks to improve the diagnostic performance of early-stage dementia.

## 3. Materials and Methods

**Figure 2:**
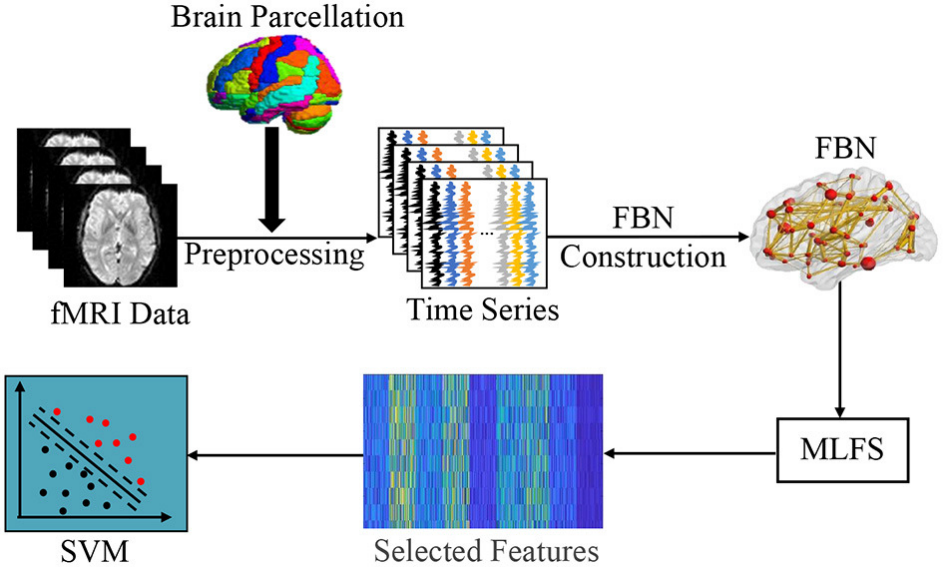
Illustration of the proposed framework for brain disease classification, including three major parts: (1) image pre-processing and FBN construction; (2) feature selection based on MLFS; and (3) classification based on support vector machine (SVM).

In this section, we first introduce the overall pipeline of FBN-based brain disease classification with the proposed MLFS method. As shown in Figure 2, this framework contains three major components, including (1) fMRI pre-processing and FBN construction; (2) feature selection based on MLFS; and (3) SVM-based classification.

### 3.1. Image Preprocessing and FBN Construction

In this paper, we evaluate our proposed method based on the dataset from the Alzheimer's Disease Neuroimaging Initiative (ADNI)^1^, which is used in a recent study (Wang et al., 2019b). The dataset contains 563 resting-state fMRI scans from 174 subjects, including 154 normal control (NC) cases, 165 early MCI (eMCI) cases, 145 late MCI (lMCI) cases, and 99 AD cases. Note that each participant may have more than one scan (with the time interval of at least 6 months between two scans). For independent evaluation, a subject-level cross-validation strategy will be used in our experiments. The scanning parameters of fMRI data are as follows: in-plane image resolution = 2.29 ~ 3.31 *mm*, slice thickness = 3.31 *mm*, echo time (TE) = 30 *ms*, repetition time (TR) = 2.2 ~ 3.1 *s*, and the scanning time for each subject is 7 *min* (resulting in 140 volumes). The demographic information of the studied subjects is summarized in Table 1.

**Table 1:** Demographic information of the involved 563 rs-fMRI scans from the ADNI database.

**Category**	**Gender (M/F)**	**Age (Years)**	**Scan #**
AD	55/44	75.04 ± 7.71	99
eMCI	73/92	72.03 ± 7.26	165
lMCI	95/50	71.99 ± 7.67	145
NC	67/87	75.36 ± 6.16	154

*The values are denoted as mean ± standard deviation. M/F, male/female*.

We process the rs-fMRI scans involved in this study by using a standard pipeline in the FSL FEAT software (Jenkinson et al., 2012). To ensure signal stabilization, the first three volumes of each subject were discarded. The remaining volumes are corrected to achieve the same slice acquisition time and remove the effect of head motion. Specifically, the subjects with the maximal translation of head motion larger than 2.0 *mm* or maximal rotation larger than 2 are excluded. Besides, the structural skull stripping is performed based on T1-weighted MRI. Then, the skull-stripped images are aligned onto the Montreal Neurological Institute (MNI) space. After all subjects were registered to the common “standard” space, the band-pass filtering is performed within a frequency interval of [0.015, 0.15 *Hz*]. Next, nuisance signals, including white matter, cerebrospinal fluid, and motion parameters, were regressed out. Then, the fMRI data are further spatially smoothed by a Gaussian kernel with full-width-at-half-maximum (FWHM) of 6 *mm*. Note that we did not perform scrubbing, since this would introduce additional artifacts. Finally, the brain space of fMRI scans is partitioned into 116 pre-defined regions-of-regions (ROIs) using the Automated Anatomical Labeling (AAL) template (Tzourio-Mazoyer et al., 2002) via a deformable registration method (Vercauteren et al., 2009). The BOLD signals from the gray matter tissue are extracted, and the mean time series of each ROI is calculated.

After image preprocessing, we use the pairwise Pearson's correlation (PC) of the extracted BOLD signals to measure the functional connectivity between each pair of ROIs. As a result, we can obtain the estimated FBN for each subject, where each node corresponds to a specific ROI and each edge weight denotes the Pearson's correlation coefficient between BOLD signals associated with a pair of ROIs. Also, we apply Fisher's r-to-z transformation to normalize the edge weights in each FBN. Note that each FBN is a singed graph, where the positive edge weights may indicate the mutual promotion and those negative edge weights may indicate the mutual inhibition (Parente et al., 2018).

### 3.2. Modular-LASSO Feature Selection

**Figure 3:**
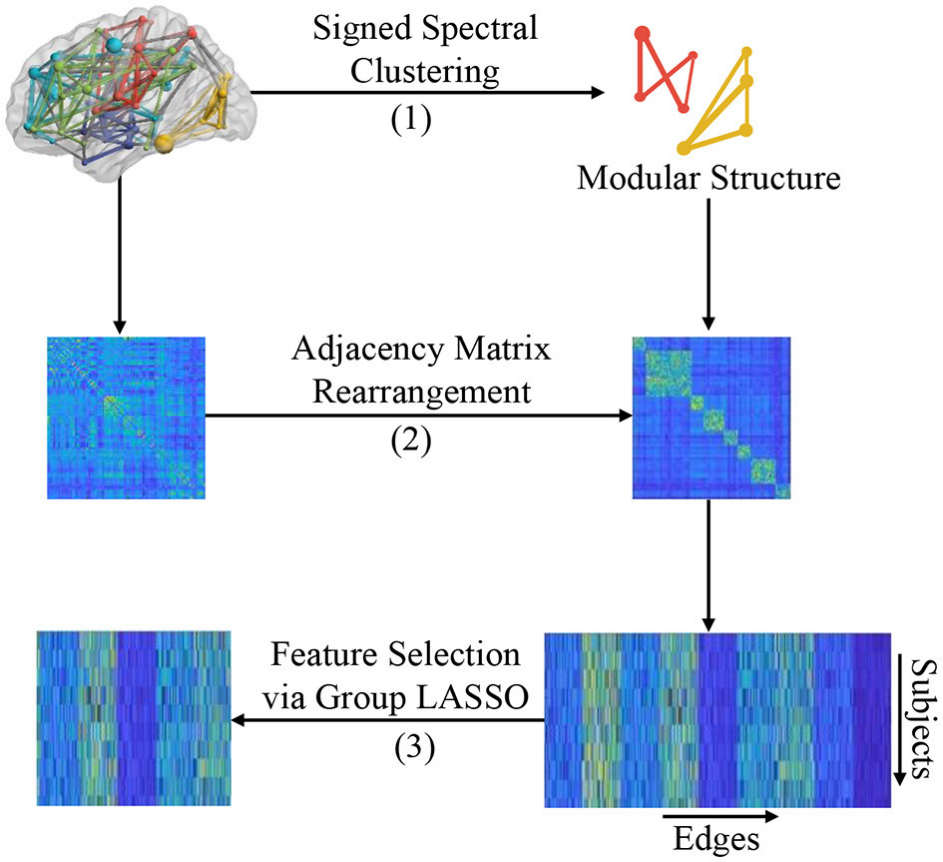
Illustration of the proposed MLFS framework that includes three major parts: (1) modular structure extraction based on signed spectral clustering, (2) adjacency matrix rearrangement based on the extracted modular structure, and (3) feature selection based on group LASSO.

In this section, we introduce the proposed modular-LASSO feature selection (MLFS) scheme for selecting features from the estimated FBNs. As shown in Figure 3, the MLFS contains three major parts: (1) modular structure extraction via a signed spectral clustering algorithm, (2) network rearrangement based on the extracted modular information, and (3) modular structure induced feature selection via group LASSO.

#### 3.2.1. Modular Structure Extraction

Nodes in an FBN tend to be organized with a modular structure, which means that nodes in the same module are densely connected with each other, and nodes of different modules are sparsely connected (Bechtel, 2003). In practice, one can employ spectral clustering algorithms to detect the modular structure in a network (Ng et al., 2002), but traditional spectral clustering methods require the adjacency matrix of a graph/network to be unsigned. Therefore, we cannot directly apply conventional spectral clustering algorithms to signed FBNs for modular structure discovery. To address this issue, a signed spectral clustering algorithm (Gallier, 2016) is used to search modular structures from singed FBNs in this work. Note that we only use the FBNs of normal controls to identify brain network modules, so as to make the identified modules more reasonable.

Denote *m* (*m* = 116 in this work) as the number of ROIs and *K* as the number of clusters (i.e., modules). An FBN is represented by an undirected weighted graph *G*(*V, E, W*) where *V* indicates the node set (i.e., ROIs), *E* indicates the edge set (i.e., functional connectivities between paired ROIs), and *W* ∈ *R*^*m*×*m*^ is the graph adjacency matrix estimated by PC. For any *i, j* ∈ *V* (*i, j* = 1, ⋯ , *m*), *w*_*ij*_ is the weight between a pair of nodes *i* and *j*. The signed degree of the node *i* is defined as follows:

(1) $$\begin{array}{r} \begin{array}{c} d_{i}=\overset{m}{\underset{j=1}{\sum }}|w_{ij}|, \end{array} \end{array}$$

and the signed degree matrix *D* ∈ *R*^*m*×*m*^ is defined as:

(2) $$\begin{array}{r} \begin{array}{c} D=diag\left(d_{i},\cdots \;,d_{m}\right). \end{array} \end{array}$$

Accordingly, the signed normalized Laplacian *L* is defined as follows:

(3) $$\begin{array}{r} \begin{array}{c} L=I-D^{-1/2}WD^{-1/2}. \end{array} \end{array}$$

Given a partition (*A*_1_, ⋯ , *A*_*K*_) of *V* (with *K* clusters), the signed normalized cut *sNcut*(*A*_1_, ⋯ , *A*_*K*_) (Gallier, 2016) is defined as follows:

(4) $$\begin{array}{r} \begin{array}{c} sNcut\left(A_{1},\cdots \;,A_{K}\right)=\overset{K}{\underset{k=1}{\sum }}\frac{{\left(X^{k}\right)}^{T}LX^{k}}{{\left(X^{k}\right)}^{T}DX^{k}}, \end{array} \end{array}$$

where *X*^*k*^ that contains the information of partition is an indicator vector for *A*_*k*_, and each cluster will be treated as a specific module. Minimizing the above objective function in Equation (4) is equivalent to solving a generalized eigenvalue equation. The optimization algorithm for the spectral clustering of signed graphs (i.e., FBNs) is shown in Algorithm 1.

**Algorithm 1:** Algorithm of singed spectral clustering.

**Require:** Adjacency matrix *W*, cluster/module number *K*.
**Ensure:** Partition (*A*_1_, ⋯ , *A*_*K*_) of *W*.
1: Construct the signed degree matrix *D*.
2: Construct the signed graph Laplacian matrix *L*.
3: Let λ_1_ ≤ λ_2_ ≤ ⋯ ≤ λ_*K*_ be the *K* smallest eigenvalues of *L* and *u*^1^, *u*^2^, ⋯ , *u*^*K*^ be the corresponding eigenvectors. Then, we can construct the matrix *U* = (*u*^1^, *u*^2^, ⋯ , *u*^*K*^) by stacking the eigenvectors in the column-wise manner.
4: Construct the matrix *F* based on *U* by normalizing each row of *U* to have the unit length.
5: By treating each row of *F* as a point, we cluster all rows in *F* into *K* clusters (via the *K*-means algorithm) and can obtain the final partition (*A*_1_, ⋯ , *A*_*K*_).

#### 3.2.2. Adjacency Matrix Rearrangement

Based on the modular structure identified by the signed spectral algorithm, we first rearrange the adjacency matrix *W* for each subject so that nodes belonging to the same module are adjacent to each other, as shown in Step (B) of Figure 3. We then reshape the rearranged adjacency matrix into an edge vector (removing the redundant part since the adjacent matrix is symmetric) to represent each subject. Finally, we pile up the edge vectors of all subjects into a data matrix (or design matrix) $X=\left[X_{w}X_{b}\right]\in R^{N\times d}$, where *N* is the number of subjects and *d* = *d*_*w*_ + *d*_*b*_ represents the number of total edges (i.e., connectivities).

This design matrix *X* consists of two parts: (1) $X_{w}\in R^{N\times d_{w}}$ that contains *d*_*w*_ *within-module edges* that connect nodes within the *K* modules (with each module as a specific group), and (2) $X_{b}\in R^{N\times d_{b}}$ that contains *d*_*b*_ *between-module edges* that connect these *K* modules and these edges can be divided into *d*_*b*_ groups (with each edge corresponding to an individual group). That is, these *d* dimensional features can be divided into *G* = *K* + *d*_*b*_ groups. In this way, each subject can be represented by both the within-module edge-level features and the between-module edge-level features of its FBN.

#### 3.2.3. Modular Structure Induced Feature Selection

We further develop a modular structure induced feature selection method to select the most informative edge-level features from FBNs for AD-related disease identification based on the group LASSO algorithm (Jiang et al., 2019). As mentioned before, *X* ∈ *R*^*N*×*d*^ is the new design matrix for *N* training samples, and *d* have been naturally divided into *G* groups. Denote *d*_*g*_ as the number of elements in the *gth* (*g* = 1, ⋯ , *G*) group, and $Y={\left[y_{1},y_{2},\cdots \;,y_{N}\right]}^{T}\in R^{N}$ as the response vector, where *y*_*i*_ (*i* = 1, ⋯ , *N*) represents the class label of the *i*^*th*^ subject. The proposed modularity-induced feature selection method can be formulated as

(5) $$\underset{\omega }{\min}\;\;\frac{1}{2}\|Y-X\omega {\|}_{2}^{2}+\lambda \sum_{g=1}^{G}\sqrt{d_{g}}\|\omega_{g}{\|}_{2},$$

where λ > 0 is the regularization parameter, and ω is the to-be-learned weighted vector which is divided into *G* groups (with ω_*g*_ representing the coefficient corresponding to the *gth* group). The second term in Equation (5) can generate a sparse solution and encourage some groups of ω to be zeros, which helps us select those edge-level features with non-zero coefficients in ω. In this way, our extracted modular structure can be explicitly employed to help identify the most informative edges in FBNs. We use the SLEP toolbox^2^ to solve the optimization problem defined in Equation (5).

### 3.3. Classification

Based on the selected features, we use a linear SVM with the default parameter (i.e., C = 1) for AD/MCI identification and MCI conversion prediction due to the two following considerations.

(1) The main goal of our experiment is to verify the effectiveness of the proposed MLFS feature selection method. However, considering the influence of different steps in the classification pipeline on the final results, it is difficult to conclude which step (FCN estimation, feature selection, and classifier) contributes more to the final accuracy. Therefore, we used the simplest and most popular classification method.
(2) It is challenging for some complicated deep learning methods, such as RCNN (Liang and Hu, 2015), BrainNetCNN (Kawahara et al., 2017), and GraphCNN (Defferrard et al., 2016), to tune hyper-parameters and train a good model without sufficient training samples (subjects). In practice, recent studies have shown that the classical machine learning algorithms tend to perform better than the deep neural networks (Dadi et al., 2019; Pervaiz et al., 2020).

## 4. Experiments

### 4.1. Competing Methods

**Figure 4:**
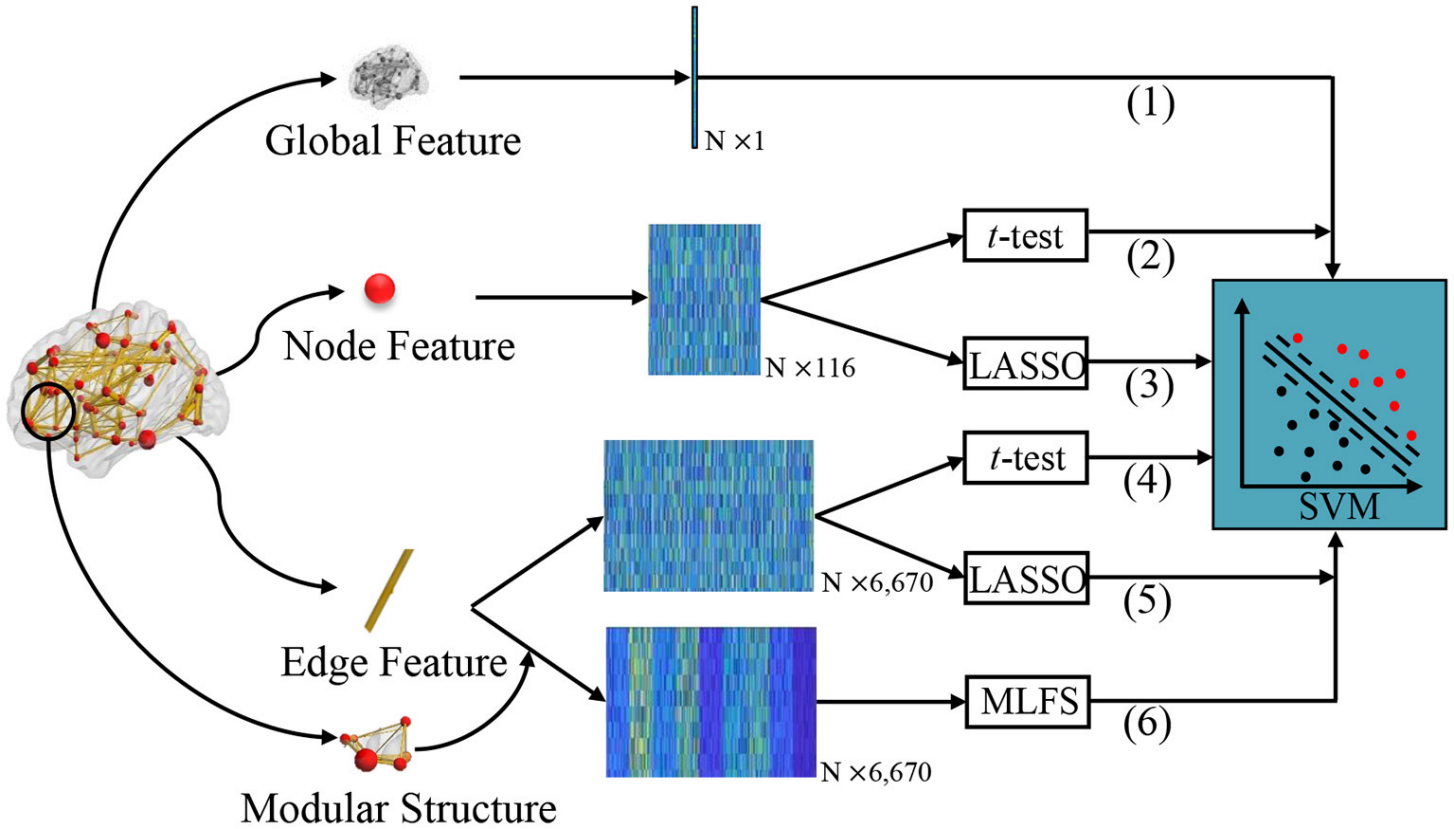
Different schemes for comparison. (1) The Global method extracts global clustering coefficients from FBNs as a one-dimensional vector. We extract local clustering coefficients from 116 nodes as a 116-dimensional matrix and perform feature selection via (2) *t*-test (called Note-*t*-test) and (3) LASSO (called Node-LASSO). We extract 116 × 115/2 network edge weights from 116 nodes as a 6,670-dimensional matrix, and perform feature selection via (4) *t*-test (called Edge-*t*-test) and (5) LASSO (called Edge-LASSO); (6) The proposed MLFS scheme for modularity-guided feature selection. For the fair comparison, the same SVM classifier is used for these six methods for classification.

In the experiments, we compare our proposed **MLFS** scheme with several traditional schemes for FBN-based classification. As shown in Figure 4, according to the different granularity, we first extract different commonly-used statistics of FBN as features, including global clustering coefficient, local clustering coefficient, and edge weights. Then, two popular feature selection algorithms, i.e., *t*-test and LASSO, are used to select discriminative features, followed by the SVM classifier. That is, the proposed MLFS is compared with five competing schemes, including (1) Global, (2) Node-*t*-test, (3) Node-LASSO, (4) Edge-*t*-test, and (5) Edge-LASSO. For a fair comparison, we employ the LIBSVM toolbox provided in Chang and Lin (2011) for SVM-based brain disease classification for all competing methods.

### 4.2. Experimental Settings

Three classification tasks are performed to evaluate the performance of our proposed method and five competing methods, including (1) MCI conversion prediction (i.e., lMCI vs. eMCI classification), (2) eMCI vs. NC classification, and (3) AD vs. NC classification. Considering the fact that one subject may have multiple scans in the dataset, using scan-level cross-validation (CV) will cause potential bias in classification. Therefore, we employ a five-fold subject-level CV strategy to ensure that the training data and test data are independent. Specifically, we first divide 174 subjects into five-fold (with each fold containing the roughly same number of subjects). Then, we use four-fold as training data to select features and train the classifier, and the remaining one-fold to validate classification performance.

**Figure 5:**
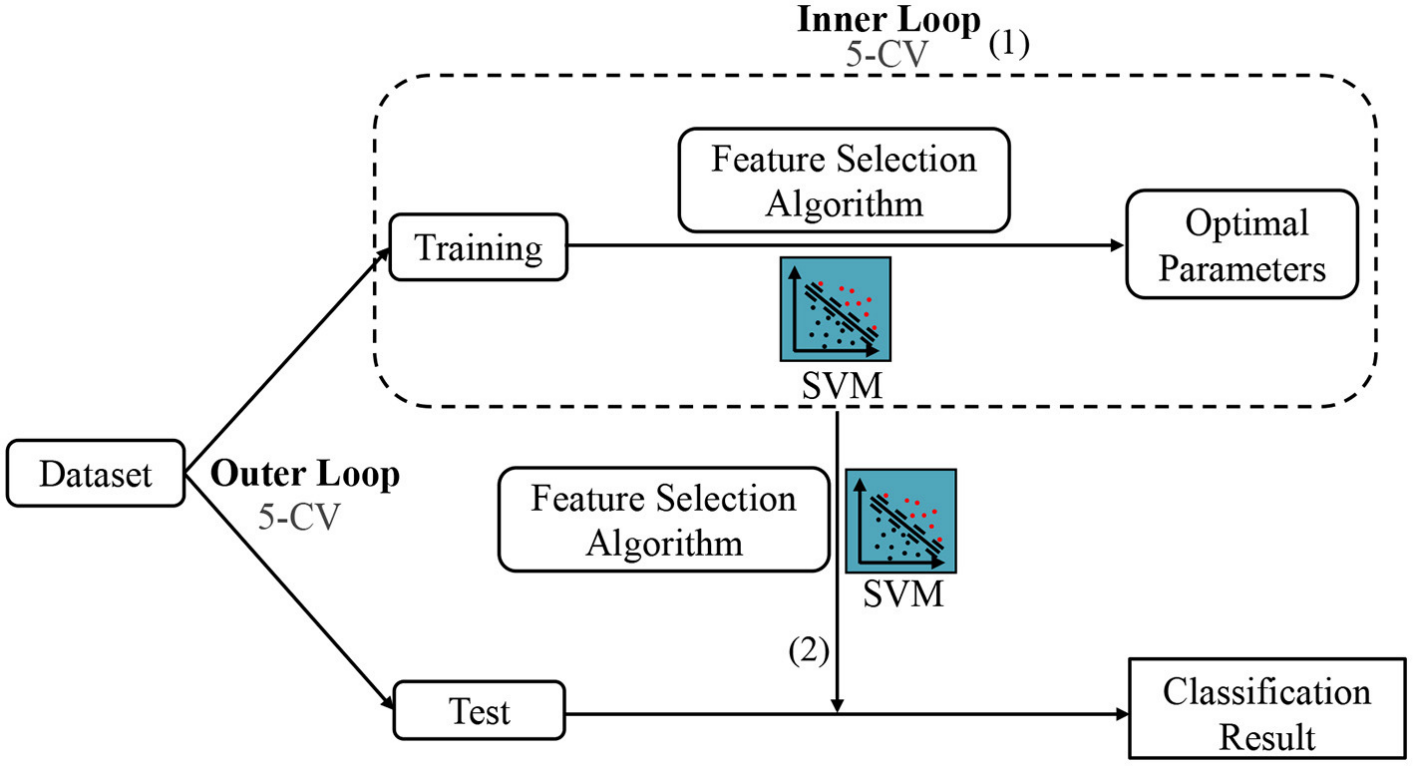
The mechanism of cross-validation in our experiment, including the Inner 5-CV to determine the optimal parameters and the outer 5-CV to get the classification results.

Besides, since the parameters involved in feature selection models may affect the number of selected features and the ultimate classification results, we conduct an inner five-fold CV on the training data to determine the optimal parameters for all competing methods, as shown in Figure 5 (1). For each parameter, we use 11 candidate values in [0.01, 0.1, 0.2, ⋯ , 0.9, 1]. Note that the optimal parameters may vary with different training sets. Therefore, we re-select features and re-train classifier (also linear SVM with C = 1) based on the current training set with optimal parameters, as shown in Figure 5 (2). Finally, we classify the test sample using the selected features and trained classifier. To avoid any bias introduced by random partition in CV, the process of data partition and five-fold CV are independently repeated 1,000 times, and the mean and standard deviation of classification results are reported. Besides, to illustrate the result is statistically significant, we perform paired t-tests (with *p* < 0.05) on the results of the involved methods, and then use the term marked by “*” to denote that the result of MLFS is significantly better than five competing methods.

We evaluate the performance of different methods via four evaluation metrics, including (1) accuracy (ACC) which is the proportion of subjects that are correctly classified samples in all samples, (2) sensitivity (SEN) which denotes the proportion of patients that are correctly classified, (3) specificity (SPE) which is the proportion of NCs that are correctly predicted, and (4) the area under the receiver operating characteristic (ROC) curve (AUC).

### 4.3. Classification Results

**Figure 6:**
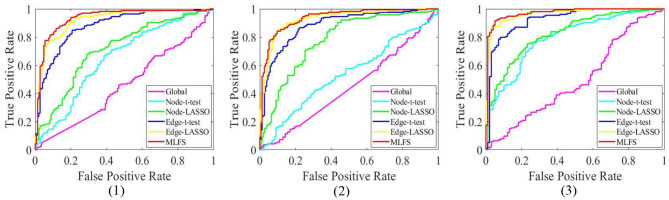
The ROC curves achieved by all six methods in three classification tasks: (1) lMCI vs. eMCI, (2) eMCI vs. NC, and (3) AD vs. NC.

Table 2 summarizes the results of six methods in three classification tasks, and Figure 6 plots the corresponding ROC curves. From Table 2 and Figure 6, we have the following interesting observations.

(1) The proposed MLFS method achieves the significant best performance in three classification tasks, compared with five competing methods. Note that the five competing methods do not consider the modularity information in FBNs. These results imply that using modularity information to guide the feature selection (as we do in MLFS) helps boost the classification performance for AD and MCI.
(2) Regarding three different granularity features (i.e., global-level, node-level, and edge-level), we can see that the performance of the Global method (based on global feature) is the worst. Also, methods using edge-level features (i.e., Edge-*t*-test, Edge-LASSO) usually outperform two methods with node-level features (i.e., Node-*t*-test, Node-LASSO). The possible reason is that edge-level features may be able to capture more topological information of FBNs and tend to result in more stable performance.
(3) Regarding three feature selection algorithms, methods with LASSO generally achieve better performance than those with *t*-test in three tasks. This may be because that *t*-test only considers the category-level differences of features and does not fully consider the relationship between features and category labels.
(4) In the task of lMCI vs. eMCI classification, the six methods achieve worse performance when compared with the other two tasks (i.e., eMCI vs. NC and AD vs. NC classification). This implies that identifying late MCI subjects from early MCI subjects is very challenging, while identifying subjects with AD/eMCI from normal controls is relatively easier. The underlying reason is that the brain function degeneration in AD and late MCI subjects could be more serious than in the early stage of MCI and NC.

**Table 2:** Classification performance of six schemes in three classification tasks (mean ± standard deviation).

**Task**	**Method**	**ACC (%)**	**SEN (%)**	**SPE (%)**	**AUC (%)**
	Global	44.52 ± 1.99	49.44 ± 2.73	47.50 ± 2.51	46.25 ± 3.81
	Node-*t*-test	59.63 ± 1.94	64.82 ± 3.36	48.31 ± 4.88	65.07 ± 2.48
	Node-LASSO	65.42 ± 1.47	78.76 ± 2.83	50.87 ± 2.50	71.43 ± 1.98
lMCI vs. eMCI	Edge-*t*-test	75.03 ± 2.64	77.52 ± 2.86	72.50 ± 5.53	87.86 ± 2.09
	Edge-LASSO	83.00 ± 1.39	85.70 ± 1.97	80.29 ± 1.62	91.89 ± 1.11
	MLFS (Ours)	84.06 ± 1.72^*^	**85.73 ± 1.84**	82.36 ± 2.39^*^	93.35 ± 1.28^*^
	Global	46.39 ± 1.98	60.00 ± 2.50	50.00 ± 2.67	44.90 ± 2.70
	Node-*t*-test	50.38 ± 2.39	71.84 ± 3.91	60.19 ± 4.47	53.74 ± 3.23
	Node-LASSO	71.38 ± 2.14	75.79 ± 2.41	67.32 ± 2.24	78.04 ± 1.56
eMCI vs. NC	Edge-*t*-test	75.23 ± 2.27	78.07 ± 3.27	77.11 ± 3.25	80.56 ± 1.85
	Edge-LASSO	84.17 ± 1.43	**85.57 ± 1.79**	83.11 ± 2.18	92.85 ± 0.99
	MLFS (Ours)	**84.80 ± 1.41**	83.74 ± 1.80	85.82 ± 2.19^*^	94.05 ± 0.98^*^
	Global	56.48 ± 1.16	61.10 ± 4.23	58.51 ± 4.50	51.14 ± 4.40
	Node-*t*-test	70.31 ± 1.78	77.41 ± 2.54	54.29 ± 5.13	79.33 ± 1.83
	Node-LASSO	74.27 ± 1.28	82.15 ± 1.69	62.33 ± 3.02	82.41 ± 1.80
AD vs. NC	Edge-*t*-test	83.44 ± 1.10	88.19 ± 1.57	76.68 ± 1.64	92.56 ± 0.62
	Edge-LASSO	88.85 ± 1.51	91.77 ± 1.15	84.16 ± 3.92	96.60 ± 0.39
	MLFS (Ours)	90.27 ± 1.02^*^	92.64 ± 1.27^*^	86.59 ± 2.38^*^	97.15 ± 0.53^*^

*Bold values indicate the best results in each task*.

## 5. Discussion

In this section, we first analyze the effect of several key hyperparameters in the proposed method, the impact of different node-level features on classification performance and the effect of connections variations in FBN. We then visualize the most discriminative features (i.e., functional connections) and modules identified by our method in different classification tasks. We also present the limitations of this work as well as several future research directions.

### 5.1. Effect of Number of Modules

**Figure 7:**
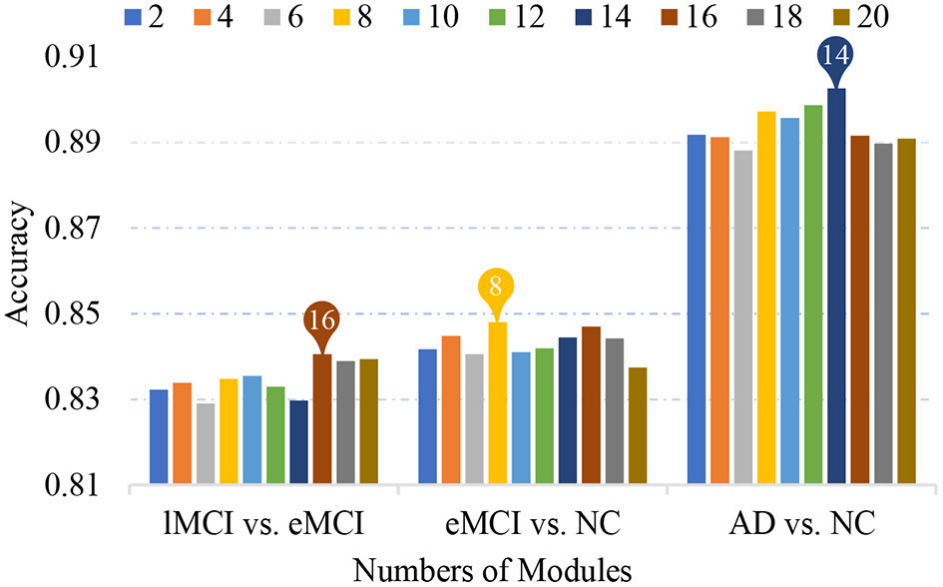
Classification accuracy achieved by the proposed method using different numbers of modules in three classification tasks.

Previous studies have found that human FBNs have a hierarchical modular organization and have different numbers of modules in each hierarchy (He et al., 2009; Meunier et al., 2009a; Power et al., 2011; Rubinov and Sporns, 2011). In our proposed MLFS scheme, we extract a total of *K* modules by using a signed spectral clustering algorithm, and the number of modules would affect the selected features and further affect classification performance. In Figure 7, we show the accuracies achieved by our MLFS in three classification tasks with respect to different numbers of modules. It can be observed from Figure 7 that, for each specific task, the accuracy values achieved by MLFS slightly vary when using different numbers of modules. And the best results are achieved when using 16, 8, and 14 modules in the task of lMCI vs. eMCI, eMCI vs. NC, and AD vs. NC classification, respectively.

### 5.2. Sensitivity to Model Parameters

**Figure 8:**
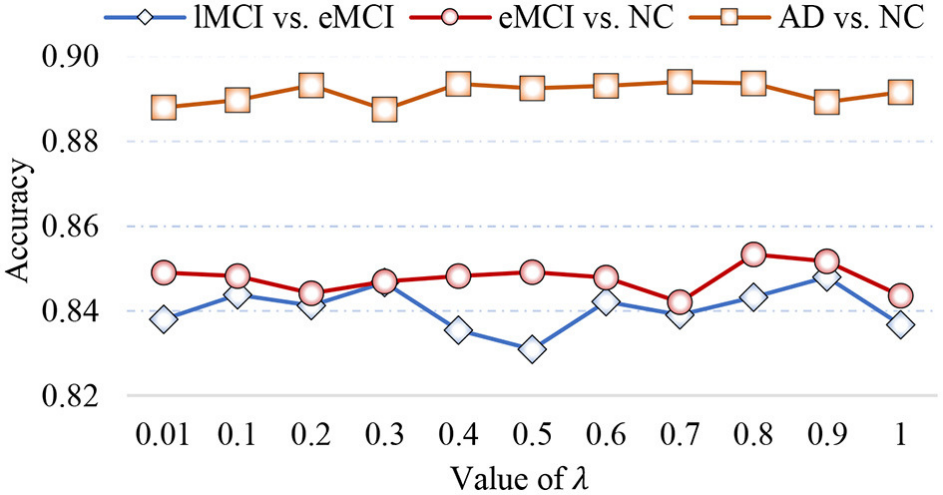
Classification accuracy achieved by the proposed method using different values of λ in three classification tasks.

In Equation (5), the parameter λ is involved in group LASSO, which may affect the number of selected features. With the optimal module numbers (i.e., 16 modules for lMCI vs. eMCI classification, 8 modules for eMCI vs. NC classification, and 14 modules for AD vs. NC classification), we calculate the classification accuracy of the proposed MLFS with different values of λ, with experimental results reported in Figure 8. As shown in Figure 8, the MLFS works well with overall stable performance in both tasks of eMCI vs. NC and AD vs. NC classification. In the task of lMCI vs. eMCI classification, the accuracy results slightly fluctuate with different values of λ. Thus, we propose to select the optimal parametric values via inner cross-validation on the training data.

### 5.3. Effect of Different Node-Level Features

**Figure 9:**
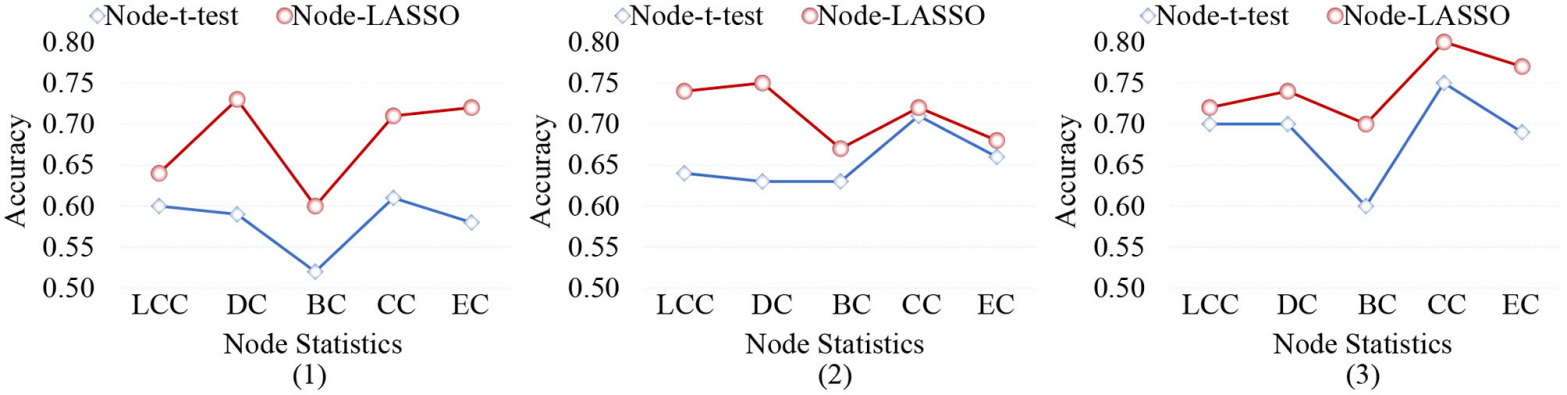
Classification accuracy achieved by the node-level methods using different node statistics in three tasks of (1) lMCI vs. eMCI, (2) eMCI vs. NC, and (3) AD vs. NC classification. LCC, local clustering coefficient; DC, degree centrality; BC, betweenness centrality; CC, closeness centrality; EC, eigenvector centrality.

When representing FBNs, node-level features can specifically locate disease-related regions, so as to help us understand the pathological mechanism of brain disorders. However, different node-level statistics extracted from FBNs tend to capture different network properties. Therefore, it is essential to analyze the effect of different node-level statistics on the final classification results. In Figure 9, we calculate the classification accuracy of the node-*t*-test method and node-LASSO method with five different node statistics: (1) local clustering coefficient (LCC), (2) degree centrality (DC), (3) betweenness centrality (BC), (4) closeness centrality (CC), and (5) eigenvector centrality (EC). It can be observed that the performance of different node-level statistics may vary for different tasks or feature selection methods. The results based on DC and CC statistics are overall the best.

### 5.4. Discriminative Connections and Brain Regions

**Figure 10:**
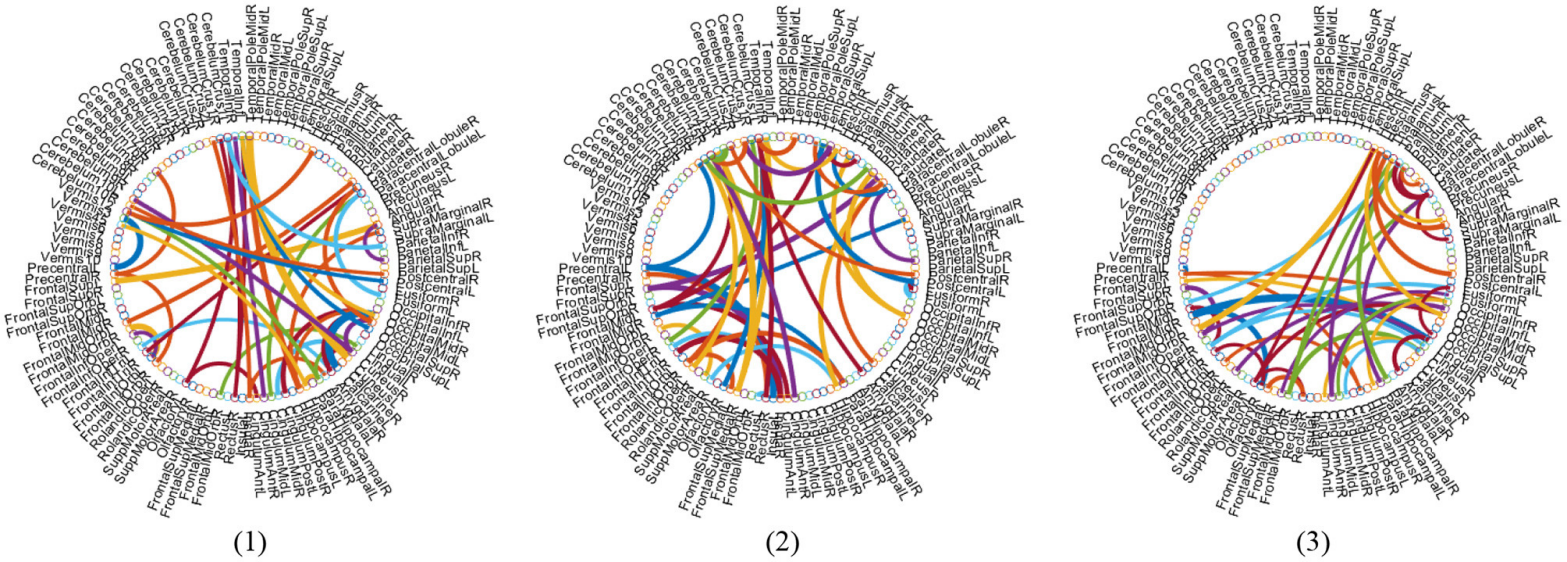
Most discriminative functional connections in three classification tasks: (1) lMCI vs. eMCI, (2) eMCI vs. NC, and (3) AD vs. NC classification.

With the empirically optimal module numbers (see Figure 7) and feature selection parameter (see Figure 8), we investigate which features are selected by the proposed MLFS scheme for AD-related disease classification. Since features selected in each fold of cross-validation could be different, we select those features that occur in all five-fold as the most discriminative features for classification. Figure 10 shows the most discriminative connections selected by MLFS in three tasks. In Figure 10, the color of each arc is randomly assigned for better visualization, and the thickness of each arc represents the discriminating power of the corresponding connection (rather than the actual connectivity strength).

**Figure 11:**
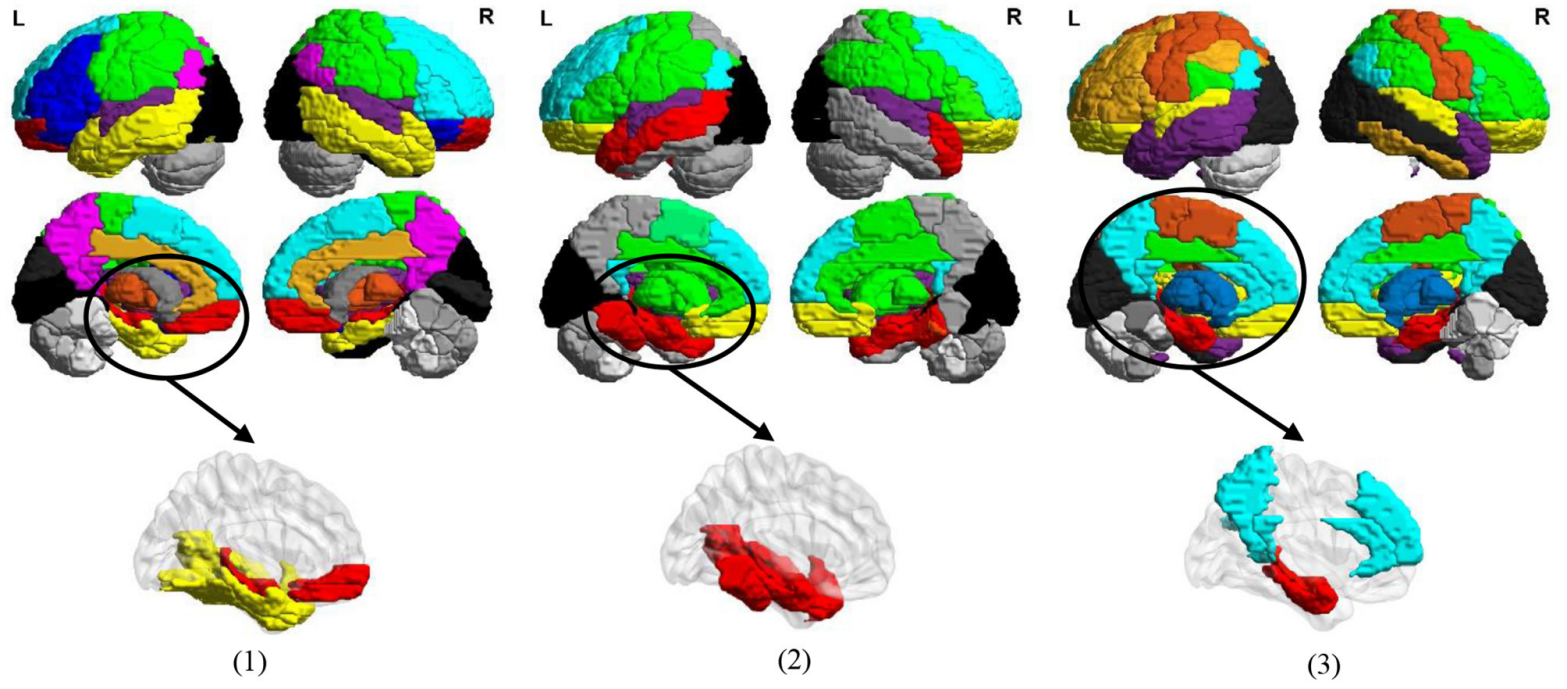
Most discriminative modules identified by the signed spectral clustering algorithm (1st and 2nd rows) and our proposed MLFS method (3rd row) based on the selected discriminative connections in three tasks of (1) lMCI vs. eMCI, (2) eMCI vs. NC, and (3) AD vs. NC classification.

In Figure 11, we visualize the modules identified by our method with the signed spectral clustering algorithm (see the 1st and 2nd rows) on the AAL template, and also visualize the most discriminative modules (see the 3rd row) based on the selected discriminative connections by our MLFS method. From this figure, we can observe that our identified discriminative modules contain several important brain regions, such as the middle temporal gyrus, hippocampus, para hippocampus, superior medial frontal gyrus, medial orbitofrontal gyrus, supramarginal gyrus and the precuneus, which have been reported in previous AD-related studies (Zhou et al., 2008; Han et al., 2012; Liu et al., 2012). These results further validate the reliability of our MLFS in identifying biomarkers for AD/MCI diagnosis.

### 5.5. Effect of Connections Variations in FBN

**Figure 12:**
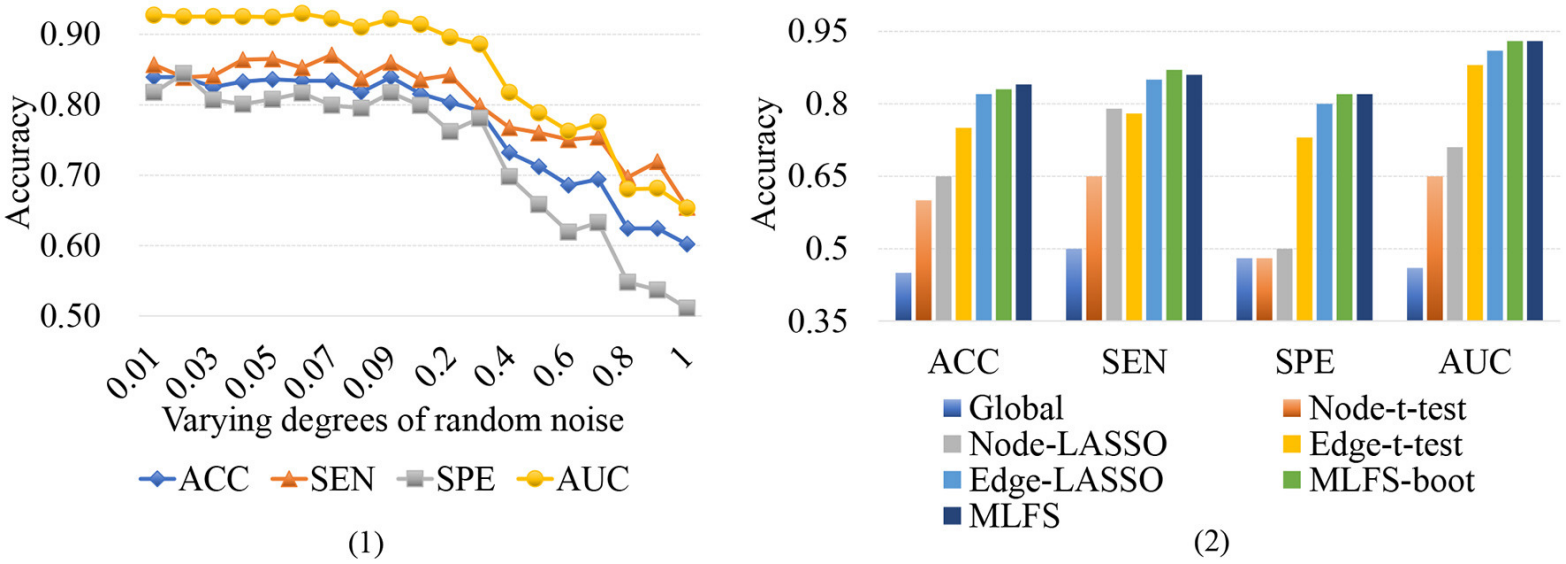
(1) Results achieved by the proposed method with varying degrees of FBN random noise in lMCI vs. eMCI classification. (2) Results achieved by the proposed MLFS-boot method, the MLFS methods, and other five competing methods in lMCI vs. eMCI classification.

Functional connectivity networks constructed via Pearson's correlation (PC) may be sensitive to noise. To investigate whether the variations of connections will influence our proposed method, we conduct a group of experiments by adding varying degrees of white Gaussian random noise to the FBN estimated by PC, and present the experimental results in Figure 12 (1). It can be observed that the classification results only show a slight fluctuation when the noise degree (standard deviation) is <0.1. However, the classification accuracy will be greatly reduced with the increase of noise degree.

To further investigate the robustness of our method, we use a standard bootstrapping process for creating several training sets (with the same size as the original training set). Then we perform the training process on these pseudo-sets and create an ensemble of classifiers. Figure 12 (2) shows the experimental results in the task of lMCI vs. eMCI classification, involving the original MLFS method, MLFS with the bootstrapping process (called MLFS-boot), and five competing methods. It can be observed from Figure 12 (2) that the proposed method outperforms five competing methods. Especially, the MLFS-boot method results in a similar performance to the MLFS method, implying that the MLFS scheme has relatively good robustness.

### 5.6. Effect of Different Network Construction Methods

In the previous experiments, we only used the Pearson's correlation algorithm for estimating FBNs, since our main focus is to use the modularity information for selecting discriminative and interpretable features. To investigate how our proposed method is affected by different network construction methods, we also use sparse inverse covariance (SIC) (Huang et al., 2010), a popular computation scheme of partial correlation, to estimate FBNs. Based on the FBNs estimated via SIC, we then conduct lMCI vs. eMCI classification and report the results of the proposed method and five competing methods in Table 3.

**Table 3:** Classification performance of six schemes in MCI conversion prediction (i.e., lMCI vs. eMCI classification) using sparse inverse covariance to estimate the FBN (mean ± standard deviation).

**Method**	**ACC (%)**	**SEN (%)**	**SPE (%)**	**AUC (%)**
Global	46.42 ± 2.09	45.23 ± 1.23	47.21 ± 1.85	46.54 ± 2.23
Node-*t*-test	62.47 ± 2.53	69.29 ± 3.10	55.47 ± 4.30	69.78 ± 2.21
Node-LASSO	65.06 ± 2.03	73.90 ± 2.87	55.55 ± 1.97	70.84 ± 1.25
Edge-*t*-test	83.75 ± 1.52	86.48 ± 2.13	80.98 ± 2.24	94.56 ± 2.09
Edge-LASSO	85.97 ± 1.72	86.07 ± 2.15	86.11 ± 2.20	93.90 ± 2.12
MLFS (Ours)	86.95 ± 1.74^*^	87.42 ± 2.03^*^	**86.71 ± 1.75**	95.16 ± 1.84^*^

*Bold values indicate the best results in each task*.

From Table 3, we have several observations that are similar to the previous experiments. First, the proposed MLFS method achieves the statistically significant best performance in lMCI vs. eMCI classification, compared with five competing methods. This indicates that our method can achieve the best performance no matter what kind of brain network estimation algorithm is used. Second, the performance of the global method (based on global feature), as always, is the worst. The edge-based methods usually outperform the node-based methods. And the methods with LASSO generally achieve better performance than those with *t*-test.

Furthermore, from Tables 2, 3, we can see that, with the same experimental settings, using SIC to estimate FBNs can get better classification performance than PC. This implies that FBNs estimated by SIC may have several advantages. On the one hand, SIC can effectively reveal the partial correlation between brain regions. That is, the FBN estimated with SIC can factor out the contribution to the pairwise correlation that might be due to global or third-party effects. This may result in clearer modules in FBN. On the other hand, SIC estimation imposes a “sparsity” constraint on the FBN, which is appropriate to model brain connectivity because many past studies based on anatomical brain databases have shown that the true brain network is sparse.

### 5.7. Limitations and Future Work

There are several limitations in the current work. *First*, we perform modular structure search and feature selection through two separate steps, so that the identified modular structures are not necessarily optimal for the subsequent classification task. As a future work, we plan to explore a joint learning framework to perform modular structure search and feature selection for FBN analysis. *Second*, only the ADNI dataset (with a limited number of fMRI scans) is used for performance evaluation in the current study. We will apply the proposed method to identify other types of brain disorders based on large-scale datasets such as ABCD (Bjork et al., 2017), ABIDE (Heinsfeld et al., 2018), and REST-meta-MDD (Yan et al., 2019). *Besides*, when constructing functional brain networks, we ignore the temporal information in the time-series data. It is interesting to employ data-driven methods (e.g., deep neural networks) to incorporate temporal dynamics into FBN construction (Wang et al., 2019b; Jie et al., 2020), which will be our future work.

## 6. Conclusion

In this paper, we propose a modularity-guided functional brain network (FBN) analysis method, namely MLFS, to identify discriminative and interpretable features from FBNs for automated AD/MCI classification. Specifically, we first search modular information of FBN by a signed spectral clustering algorithm and then select edge-level network features based on a modularity-induced group LASSO method. Finally, we use the selected features to identify different stages of subjects with AD or MCI. Experimental results on 563 rs-fMRI scans from ADNI suggest the superiority of the proposed method in three classification tasks, compared with conventional methods for FBN-based brain disease diagnosis.

## Data Availability Statement

The original contributions presented in the study are included in the article/supplementary material, further inquiries can be directed to the corresponding author/s.

## Ethics Statement

The studies involving human participants were reviewed and approved by the open dataset of Alzheimer's Disease Neuroimaging Initiative. The patients/participants provided their written informed consent to participate in this study. Written informed consent was obtained from the individual(s) for the publication of any potentially identifiable images or data included in this article.

## Author Contributions

YZ and LQ designed the study. YZ downloaded and analyzed the data, performed experiments, and drafted the manuscript. YZ, XJ, LQ, and ML revised the manuscript. All the authors read and approved the final manuscript.

## Conflict of Interest

The authors declare that the research was conducted in the absence of any commercial or financial relationships that could be construed as a potential conflict of interest.

## Publisher's Note

All claims expressed in this article are solely those of the authors and do not necessarily represent those of their affiliated organizations, or those of the publisher, the editors and the reviewers. Any product that may be evaluated in this article, or claim that may be made by its manufacturer, is not guaranteed or endorsed by the publisher.

## Footnotes

^1^ http://adni.loni.usc.edu/

^2^ http://yelabs.net/software/SLEP/
